# Cardiac Pathophysiology and the Future of Cardiac Therapies in Duchenne Muscular Dystrophy

**DOI:** 10.3390/ijms20174098

**Published:** 2019-08-22

**Authors:** Tatyana A. Meyers, DeWayne Townsend

**Affiliations:** Department of Integrative Biology and Physiology, Medical School, University of Minnesota, Minneapolis, MN 55455, USA

**Keywords:** Duchenne muscular dystrophy, dystrophic cardiomyopathy, fibrosis, gene therapy, exon skipping, heart disease

## Abstract

Duchenne muscular dystrophy (DMD) is a devastating disease featuring skeletal muscle wasting, respiratory insufficiency, and cardiomyopathy. Historically, respiratory failure has been the leading cause of mortality in DMD, but recent improvements in symptomatic respiratory management have extended the life expectancy of DMD patients. With increased longevity, the clinical relevance of heart disease in DMD is growing, as virtually all DMD patients over 18 year of age display signs of cardiomyopathy. This review will focus on the pathophysiological basis of DMD in the heart and discuss the therapeutic approaches currently in use and those in development to treat dystrophic cardiomyopathy. The first section will describe the aspects of the DMD that result in the loss of cardiac tissue and accumulation of fibrosis. The second section will discuss cardiac small molecule therapies currently used to treat heart disease in DMD, with a focus on the evidence supporting the use of each drug in dystrophic patients. The final section will outline the strengths and limitations of approaches directed at correcting the genetic defect through dystrophin gene replacement, modification, or repair. There are several new and promising therapeutic approaches that may protect the dystrophic heart, but their limitations suggest that future management of dystrophic cardiomyopathy may benefit from combining gene-targeted therapies with small molecule therapies. Understanding the mechanistic basis of dystrophic heart disease and the effects of current and emerging therapies will be critical for their success in the treatment of patients with DMD.

## 1. Introduction

Muscular dystrophies are a diverse group of rare genetic diseases characterized by progressive skeletal muscle wasting. Many different molecular mechanisms underlie this loss of healthy muscle tissue, leading to wide variability in severity and affected muscle groups. Some of the more devastating muscular dystrophies are those that develop due to the absence of components of the dystrophin-glycoprotein complex (DGC), resulting in compromised sarcolemmal integrity in skeletal muscle and the heart. These forms of muscular dystrophy feature both skeletal muscle wasting and marked cardiomyopathy. One of the most common forms of muscular dystrophy is Duchenne muscular dystrophy (DMD), which arises due to mutations in the dystrophin gene that result in the complete absence of this large protein that functions in stabilizing the myocyte membrane. Without dystrophin scaffolding the cytoskeleton to the sarcolemma, the mechanical and molecular infrastructure of the cell is significantly compromised. This gives rise to a variety of downstream pathogenic mechanisms, culminating in disease characteristics that include striated muscle degeneration, fibrosis, loss of motor function, and death due to cardiorespiratory failure.

With advancements in life-prolonging symptomatic therapies like ventilatory support, a greater proportion of patients with severe forms of muscular dystrophy survive long enough to develop heart failure, increasing the need for interventions that can ameliorate heart disease in this population [1,2,3]. In the 21st century, heart failure is a leading cause of death in DMD, with many patients reaching adolescence or adulthood and displaying symptoms of dystrophic heart disease without receiving cardiac therapy [2,4]. To address this issue, the most current guidelines released in 2015 recommend that DMD patients start therapy with ACE inhibitors (ACEIs) or angiotensin receptor blockers (ARBs) by 10 years of age or sooner, based on mounting evidence that early stages of cardiac deterioration may have already begun [5,6].

Currently, there is great excitement in the muscular dystrophy community as new therapeutic approaches are reaching the clinics. While the overall efficacy of these new therapies will be measured in ongoing clinical trials, most of these trials lack detailed cardiac end-points, and some of these approaches are known to have relatively poor efficacy in the dystrophic heart. Given the critical role of the heart in the disease process and quality of life, this review will focus on current understanding of cardiac involvement in DMD and detail how current and future therapies may delay or prevent the onset of heart failure in DMD patients.

## 2. Genetic Basis

One of the earliest recognized features of DMD was its X-linked mode of inheritance, which greatly aided in the identification of the genetic locus that was responsible for the disease. The *DMD* gene is the largest known human gene at 2.4 Mb, producing a 14 Kb mRNA transcript from 79 exons. Several internal promoters and variable splicing give rise to a wide variety of dystrophin isoforms that are expressed in striated and smooth muscle, brain, retina, and kidney [7]. This vast size and complexity likely contribute to higher probability of a mutation interfering with the gene product. Deletions are the most common type of dystrophin mutation underlying DMD, accounting for over 70% of all mutations and often causing a change in the reading frame that produces a premature stop codon [8,9]. The next most common types of *DMD* mutation are insertions and point mutations, also usually resulting in a premature stop codon and the termination of protein synthesis [8,9]. DMD is most often passed down via the X-chromosome from a mother that carries one mutated copy of the gene to male offspring, but also has a relatively high rate of de novo mutations accounting for roughly 1/3 of all cases [8,9]. Because DMD is an X-linked disease, it affects almost exclusively males, with an incidence of approximately 1 in 5000 live male births [10,11].

Conversely, mutations that still allow for a truncated dystrophin to be produced and trafficked to the sarcolemma result in the overall milder Becker muscular dystrophy (BMD). With an incidence of about 1 in 18,500 male births, BMD is much rarer than DMD due to its roots in dystrophin mutations that preserve the open reading frame, allowing for a partially functional protein product [9]. BMD displays wide phenotypic variation, ranging from very severe DMD-like disease to very mild muscle weakness. This phenotypic variation depends on the specific regions of dystrophin that are lost as a result of the BMD-causing mutation [12,13]. Accordingly, analysis of the relationship between the underlying mutation and resulting phenotype in BMD has been instrumental in shaping our understanding of dystrophin’s structure and the function of its various domains. In fact, identification of some BMD-causing mutations that produced extensive deletions in the central domain but resulted in a very mild disease course has led to the development of multiple truncated but functional micro-dystrophins as gene therapy candidates currently in clinical trials.

## 3. Clinical Manifestation

Duchenne muscular dystrophy was first described in the early 19th century by Italian physicians Gaetano Conte and L. Gioja. In the 1830s, they reported on two brothers displaying progressive muscle weakness in the face of paradoxical hypertrophy, with the older brother dying with an enlarged heart, and the younger eventually losing the ability to move [14]. Brothers afflicted by debilitating muscle deterioration along with hypertrophy in the absence of neurological deficits were again described in 1852 and 1853 by Edward Meryon and William J. Little, respectively [14]. However, the most detailed account was delivered in the 1860s by Guillaume-Benjamin-Amand Duchenne, who provided photos, drawings, and detailed descriptions of 13 of his own patients who shared these characteristics of muscle wasting, progressive muscle weakness accompanied by pseudohypertrophy, and premature death [14].

Initial clinical symptoms tend to be noticed around 3–5 years of age and typically include apparent muscle weakness and fatigue in the legs and pelvic region, causing an abnormal gait, lordosis, and use of Gowers’ maneuver. The gastrocnemius eventually develops pseudohypertrophy, resulting from accumulation of fatty and fibrotic tissue combined with slower atrophy than in the thigh muscles [15]. Elevations in serum levels of creatine kinase (CK), an enzyme typically retained within muscle fibers in healthy tissue, are detectable in newborns with muscular dystrophy at levels around 10-fold higher than in healthy newborns [10]. As children with DMD grow and reach a symptomatic age, serum CK is further elevated to about 50–100 times of the healthy level as myofiber contents leak out of increasingly damaged muscle cells. Since dystrophin also plays a role in the brain and affects cognitive function, cognitive or neuropsychiatric impairment present in roughly 1/3 of all cases [16,17]. Histopathological evaluation of patient muscle biopsies reveals central nucleation indicative of ongoing muscle regeneration, along with fibrosis, myocyte necrosis, fatty accumulation, and immune cell infiltration.

Cardiac functional assessment via echocardiography shows systolic and diastolic dysfunction as the disease progresses. Declines in ejection fraction (EF) to <50% can be seen as early as 9–10 years of age, and ejection fraction may drop as low as 25–30% later in the course of the disease [18]. Cardiac magnetic resonance imaging (CMR) may be used to detect declining myocardial strain as an index of cardiac dysfunction before the onset of measurable EF deficits [19]. In combination with late gadolinium enhancement (LGE), cardiac MRI can also be used to identify fibrotic scarring in the heart, which appears ahead of cardiac dysfunction [18]. This LGE+ fibrosis tends to be localized sub-epicardially and often inferobasally, appearing in some patients even before 10 years of age and in most patients after 15 years of age, and increases in LGE are correlated with age and declining ejection fraction [7,18,20]. Cardiac troponin (cTn) elevations can be intermittently detected in patient serum, serving as a biomarker of sporadic periods of myocardial destruction [21,22,23]. Heart rhythm abnormalities are also present in a significant portion of patients, usually in the form of sinus tachycardia, and may be related to myocardial fibrosis in the cardiac conduction system [7].

As the disease progresses, atrophy of posture muscles often leads to scoliosis, and further muscle wasting and contractures result in wheelchair dependence around adolescence. Cardiac dysfunction usually progresses into dilated cardiomyopathy with extensive fibrosis, shown to be present in essentially all patients over 18 years of age, and eventually heart failure sets in [3,24]. Historically, death usually occurs by 20–25 years of age from cardiorespiratory failure, but advancements in respiratory and pharmacological interventions have the capacity to extend lifespans into the 30s and early 40s with diligent clinical management [1,25].

## 4. Pathophysiological Mechanisms of Dystrophic Cardiomyopathy

### 4.1. Membrane Instability

Membrane fragility has often been viewed as the primary defect in DMD, precipitating multiple secondary pathophysiological mechanisms that lead to myocyte death. In healthy myocytes, rather than distributing diffusely throughout the sarcolemma, dystrophin localizes in a striated pattern that reflects its association with costameres [26]. Costameres are a sub-sarcolemmal network that mechanically couples the extracellular matrix (ECM) to the Z-disc of the sarcomere through the DGC and cytoskeletal γ-actin, with dystrophin playing a central role in this mechanical linkage [27]. Dystrophin’s N-terminal region binds filamentous γ-actin and extends to the sarcolemma, where its C-terminus joins with the transmembrane β-dystroglycan. β-dystroglycan’s extracellular domain binds to the heavily glycosylated α-dystroglycan, which in turn links to the ECM through laminin. β-dystroglycan also associates with the transmembrane sarcoglycan complex, which plays its own major role in sarcolemmal stability. This assembly comprises the DGC, and this axis of force transmission and stabilization from the contractile apparatus to the ECM is broken when dystrophin is not expressed. It is worth noting that dystrophin loss precipitates the depletion of the majority of the DGC in skeletal muscle, but cardiac muscle retains most of the other DGC components in the absence of dystrophin [28,29].

Without functional dystrophin, the sarcolemma becomes fragile and displays significant leakiness under increased workload on the myocyte. Serum biomarkers of this increased cellular permeability include elevated levels of CK, the bulk of which is attributable to skeletal muscle, and cTn from the myocardium [21,22,30]. Further preclinical evidence of major membrane disruptions is readily detectable in dystrophic hearts and muscle by Evans Blue dye or endogenous IgG entry into the myocytes, indicating where the sarcolemma was compromised enough to permit influx of serum proteins. These assays reveal markedly higher susceptibility to cardiac and skeletal myocyte injury in dystrophic animals lacking dystrophin compared to healthy controls [31,32,33].

Studies using genetic models have substantiated a pivotal role for membrane integrity in cardiomyocyte preservation and dystrophic cardiomyopathy. Dysferlin, MG53, and thrombospondin 4 (Thbs4) have been identified as important players in membrane repair and maintenance. Ablation of any of these genes in dystrophin-null or sarcoglycan-null animals has been shown to worsen their cardiac and skeletal muscle pathology [34,35,36,37,38]. In fact, primary dysferlin deficiency is associated with muscular dystrophy in animal models and human patients (Miyoshi Myopathy and LGMD2B) characterized by mild-moderate muscle weakness and infrequent cardiac dysfunction [39]. On the other hand, overexpression or exogenous delivery of MG53 or Thbs4 have been shown to attenuate the severity of myocyte pathology in dystrophic animal models [37,40,41].

In an effort to limit muscular dystrophy and dystrophic cardiomyopathy by patching the sarcolemma, multiple groups have turned to synthetic amphiphilic copolymers, chiefly poloxamer 188 (P188), as a potential therapy. P188 works to reduce leak in biological membranes by partitioning into the hydrophobic region exposed when the lipid bilayer tears, as demonstrated by molecular simulations [42,43]. Work in cells has yielded convincing evidence of marked reductions in membrane leak following P188 treatment, and dystrophic animal studies with P188 have reinforced the concept of membrane disruption as a major driver of dystrophic pathology [44,45,46,47]. Specifically, dystrophic dogs and mdx mice showed significant amelioration of various disease indices following P188 treatment, including improved cardiac histopathology and reduced myocyte force drop. Accordingly, a human trial is currently being initiated to test the safety and efficacy of P188 therapy in DMD patients.

### 4.2. Calcium Dysregulation

A major feature of homeostatic disruption that stems from high membrane permeability is the erosion of ion gradients, which contributes to including increased intracellular calcium levels. In addition to passive calcium leak through membrane tears, a number of ion channels and calcium handling proteins may play a role in the elevations in cytosolic calcium concentrations found in dystrophic myocytes. Cardiomyocytes from mdx hearts display high diastolic calcium levels, augmented calcium influx in response to stretch, altered calcium transient kinetics, and changes in calcium-handling protein expression, activation, or post-translational modifications [45,48,49].

Aside from passive calcium entry through the leaky membrane, transient receptor potential (TRP) channels and L-type Ca^2+^ channels (LTCC) may be allowing excessive calcium entry into dystrophic cardiomyocytes [50]. TRP channels, including TRPC1, TRPC6, and TRPV2 are thought to participate in the augmented stretch-stimulated cation influx observed in mdx cells, and have been shown to be overexpressed and/or hyperactive in dystrophic myocytes [50,51,52,53]. Notably, inhibition of TRPC1 and TRPV2 showed a benefit in mdx cardiomyocytes at baseline and under osmotic stress by reducing Ca^2+^ accumulation [49,52]. Other studies have shown enhanced calcium influx through the L-type Ca^2+^ channel in cardiomyocytes from mice lacking just dystrophin or both dystrophin and utrophin, and linked this change to delayed inactivation of the LTCC [50,54,55,56].

Excess diastolic calcium found in dystrophic cardiomyocytes may also come from the sarcoplasmic reticulum (SR) in addition to extracellular sources. Ryanodine receptor 2 (RyR2) and the sarcoplasmic/endoplasmic reticulum calcium ATPase (SERCA2a) are two of the key players that may drive excessive store-operated calcium release in dystrophic cardiomyocytes. In addition to some studies documenting altered channel expression, RyR2 has also been shown to undergo altered phosphorylation and S-nitrosylation in dystrophic hearts, resulting in disrupted channel regulation by calstabin and enhanced release of Ca^2+^ from the SR [57,58,59]. Accordingly, treatments interfering with RyR phosphorylation or reinforcing its association with calstabin were shown to normalize cytosolic calcium levels, reduce arrhythmogenicity, and improve cardiac pathology in mdx hearts [58,59,60,61]. In a similar vein, some studies have demonstrated SERCA2a activity to be reduced in dystrophic hearts, possibly due to some combination of lower SECRA2a expression and alterations in its regulation by the inhibitory proteins phospholamban and sarcolipin [49,62,63]. Together, the failure of these mechanisms to adequately sequester SR calcium to maintain low diastolic calcium levels may compound upon the problem of high calcium leak across the sarcolemma, resulting in significant myocyte pathology.

Elevated intracellular calcium interferes with proper contractile and electrical activity in a myocyte and stimulates increased proteolysis, mitochondrial dysfunction, and signaling cascades that promote cell death [64]. Calpains are a family of proteases activated by calcium that have an abundance of cellular targets, including cytoskeletal proteins, receptors, ion channels, and proteins that participate in excitation-contraction coupling [65]. It has been suggested that robust activation of calpain activity could also lead to degradation of non-target proteins, and may even participate in activation of the ubiquitin-proteasome system to drive further proteolysis [66,67]. Recently it was demonstrated that, even in healthy mouse hearts, cardiomyocyte injury resulting in endogenous IgG entry into the cell coincides with loss of dystrophin and destruction of the orderly sarcomeric organization of α-actinin within eight hours after the injury [31]. These outcomes likely reflect the mass action of proteases activated by the calcium influx following major membrane disruption, but similar processes may be underway in dystrophic myocytes that have constant low-grade calcium elevations.

In addition to triggering proteolysis, accumulated cytosolic calcium also makes its way into the mitochondria, where it may induce mitochondrial dysfunction that can precipitate cell death. Mitochondrial oxidative metabolism depends upon the maintenance of a negative mitochondrial inner membrane potential that is essential for ATP synthesis. This process is further stimulated by temporary elevations in mitochondrial matrix calcium that occur during increased workloads in healthy mitochondria [68]. However, excessive calcium loading into the mitochondria may trigger opening of the mitochondrial permeability transition pore (mPTP), depolarizing the mitochondria and interrupting ATP synthesis unless the mitochondrial membrane potential can be restored [69]. Opening of the mPTP leads to mitochondrial swelling, potentially resulting in destruction of the organelle, and can ultimately trigger cell death by depleting cellular ATP content and releasing reactive oxygen species and pro-apoptotic factors from mitochondria into the cytosol [69]. Indeed, mdx cardiomyocytes have been shown to have elevated mitochondrial calcium content and mitochondrial swelling, inspiring ongoing efforts to impede mPTP opening or mitochondrial calcium uptake to improve pathology in preclinical dystrophic animal models [55,70,71].

### 4.3. Mitochondrial Energetics

The heart uses a tremendous amount of ATP to perform its physiological functions. In order to meet these very high and dynamic energy needs, the heart has evolved an energetic buffer and shuttling mechanism using phosphocreatine to store and transport chemical energy. Phosphocreatine (PCr) can serve as a fuel buffer that can be rapidly converted into ATP when cardiac energy demands increase. When energy production begins to decline, the levels of PCr decrease as more is used to maintain ATP concentrations. Therefore, reductions in PCr:ATP ratio are often used to measure the energetic reserves of the heart [72]. Preclinical studies in the mdx mouse have demonstrated that the PCr:ATP ratio is significantly reduced prior to the onset of significant cardiac fibrosis [73,74]. These observations that the dystrophic heart is energy-starved early in the course of the disease raises important questions regarding the role of this energetic decline in the pathophysiology of dystrophic cardiomyopathy. The oxygen consumption of the isolated dystrophic heart is slightly elevated relative to wild type hearts, suggesting that the overall oxidative capacity of the dystrophic heart is relatively normal [74,75]. These data are consistent with the normal levels of maximal oxygen consumption in isolated mitochondria and cardiomyocytes from dystrophic hearts [55,75]. Some reports have demonstrated that a small percentage of dystrophic mitochondria have abnormal cristae structure and dilated appearance, although other studies observed no differences in the mitochondria of dystrophic hearts [70,75]. Importantly, as the dystrophic process progresses, these deficits in mitochondrial morphology become more apparent, reflecting that mitochondrial dysfunction is potentially both a cause and effect of the dystrophic process [76].

Together, these findings demonstrate that mitochondrial energy production is limited early in the disease process, well before significant fibrosis or declines in contractile function are evident. Interestingly, carriers of DMD-causing mutations, who have dystrophin missing from roughly half of their cardiac myocytes, also display reductions in PCr:ATP ratios in the presence of normal contractile function [77]. This observation reinforces the notion that energetic declines are an important feature of dystrophic cardiomyopathy. Understanding the underlying mechanism by which dystrophic hearts become energetically limited could provide a new therapeutic approach to limit the progression of the disease.

One potential mechanism of energetic deficiency may stem from increases in ATPase activity that the mitochondria are not able to match, resulting in a decline in PCr, the energetic buffer in cardiomyocytes. The formation of tears in the membrane will result in the loss of ionic gradients, and energy needs to be expended to restore these gradients by driving membrane ATPases. It is difficult to estimate with any certainty the extent of this increase in ATPase activity, although it may be reflected in the increased baseline oxygen consumption of the dystrophic heart [74,75]. With healthy mitochondria, any increases in ADP levels should result in increases in ATP synthesis. This is not observed in the dystrophic heart, where ADP levels are significantly elevated relative to wild type hearts. This observation, coupled with reductions in PCr:ATP ratio, suggest that there is some limitation on the ATP synthesis of the mitochondria in dystrophic hearts. As noted above, in the early stages of the disease, this deficit is subtle and may only become functionally relevant with cardiac stress. However, the failure of these mitochondria to provide sufficient energy to the dystrophic heart during periods of increased cardiac workloads could have devastating effects on cellular homeostasis, thus increasing the likelihood of cardiomyocyte death.

The previous discussion argues the point that the mitochondria of the dystrophic heart are somehow defective and unable to meet the energetic demands of the dystrophic cardiomyocyte. How the loss of dystrophin results in mitochondrial dysfunction is poorly understood. The presence of elevated diastolic calcium levels in dystrophic hearts suggests the possibility that dystrophic mitochondria are overloaded with Ca^2+^. This is supported by evidence that mitochondria from mdx mice are more sensitive to Ca^2+^-induced opening of the mPTP [70,78]. The Ca^2+^-dependent opening of the mPTP is well described and occurs in forms of both catastrophic opening that can result in cell death and transient opening that results in the run-down of the mitochondrial membrane potential [79,80,81]. This will decrease the amount of ATP produced for a given oxygen consumption, resulting in a reduction in myocardial efficiency. The frequent opening of the mPTP could largely explain the reduced energetics and increased oxygen consumption observed in the dystrophic heart. Other potential mechanisms involve the disruptions of nitric oxide production or microtubule arrays [82,83,84]. The reduced capacity of dystrophic mitochondria to respond to increases in energetic demands of the myocytes may have significant implications for how dystrophic myocytes handle physiological stress. From the prolonged restoration of ionic gradients following membrane injury to the poor contractile function elicited from adrenergic stimulation, these characteristics of the dystrophic heart may have their root in limited mitochondrial capacity.

### 4.4. Reactive Oxygen Species Dysregulation

Dystrophin loss is associated with increased generation of reactive oxygen species (ROS) that contribute to the pathogenesis of DMD. A significant source of ROS production is NADPH oxidase 2 (NOX2), the primary muscle isoform of this membrane-bound superoxide-generating enzyme. Mdx mice have been consistently shown to harbor increased NOX2 expression and activity, and NOX2 appears to be responsible for exaggerated stretch-induced ROS production in both skeletal and cardiac myocytes from mdx mice [84,85]. This exaggerated increase in NOX2-dependent superoxide production appears to underlie the disorganization of microtubules in skeletal muscle, likely also contributing to the disorganized microtubule array in dystrophic cardiomyocytes [83,86,87]. Upon activation, NOX2 produces extracellular superoxide, which is converted by extracellular superoxide dismutase to the membrane-permeant H_2_O_2_ [88]. Once inside the myocyte, excess H_2_O_2_ can cause oxidation of proteins, lipids, carbohydrates, and nucleic acids, precipitating a variety of pathological effects that include deficits in calcium handling, contractility, and cell survival [89,90]. In fact, evidence suggests that NOX2-generated ROS may contribute to SR calcium leak and mitochondrial dysfunction in mdx myocytes [50,84,85,86,90,91].

Accordingly, many studies have demonstrated a protective effect of pharmacological or genetic NOX2 inhibition in mdx heart and skeletal muscle, with decreased ROS generation, improved calcium handling, increased myocyte survival, and greater force production among the associated benefits [33,61,84,85,90,91]. NOX2 is also a likely downstream effector of angiotensin II type 1 receptor (AT_1_R), which may be activated by the binding of angiotensin II or by stretch to stimulate ROS production via NOX2 [89,92,93]. This relationship could account for some of the reported benefits of angiotensin receptor blockers (ARBs) in dystrophic models, including recent work showing acute protection of mdx myocardium when treated with losartan only one hour before inducing cardiac injury with isoproterenol [31,94,95].

### 4.5. Nitric Oxide Dysregulation

In skeletal muscle, there is clear evidence that dystrophin is critical for the localization of neuronal nitric oxide synthase (nNOS) at the membrane of the muscle fiber [90,96]. From this sub-membrane compartment, NO readily diffuses out of the cell, where it signals to neighboring vascular cells to dilate the vasculature, increasing blood flow to actively contracting muscle [97,98]. The presence of this molecular arrangement in the heart is not clearly understood. While dystrophin and nNOS are both expressed in the myocardium, co-immunoprecipitation studies have demonstrated that they do not interact to the same degree as in skeletal muscle [99]. Further, there are many concurrent mechanisms controlling coronary blood flow that may render this nNOS localization less necessary.

Complicating the understanding of cardiac nitric oxide signaling is the fact that all three isoforms of NOS—nNOS, endothelial NOS (eNOS), and inducible NOS (iNOS)—are expressed in the heart. Evidence of NO-related alterations from preclinical DMD studies include declines in nNOS activity and expression and significant increases in iNOS activity and expression, but no reported change in eNOS activity and expression [58,85,100,101,102]. iNOS is of particular interest in dystrophic hearts and muscle, as its expression is typically high in immune cells, and it is further increased with inflammation, potentially resulting in a large contribution of iNOS to NO production in regions of ongoing tissue injury and degeneration [90,103,104]. Furthermore, despite the lack of evidence supporting the interaction of nNOS with dystrophin in the heart, there are indications that mitochondria from dystrophic hearts are associated with nNOS activity that is not present in wild type hearts [82]. This suggests that nNOS localization is abnormal in dystrophic cardiomyocytes, which, together with the increase in iNOS activity, results in complex effects that alter the NO-dependent physiology of the dystrophic heart. Finally, both skeletal muscle and the heart harbor eNOS, and the regulation of its activity is multifactorial and complex, creating major obstacles to understanding this isoform’s contribution to the NO metabolism of dystrophic myocytes [105].

The signaling of nitric oxide is complex, but it can be broadly divided into roughly two branches: one mediated by cyclic guanylyl monophosphate (cGMP), and another via direct covalent modifications. Initially, preclinical work suggested that the cGMP branch of NO signaling may provide significant benefit, as both genetic and pharmacological manipulations of this pathway appeared to partially improve dystrophic cardiac function in mice [106,107]. Based on the promise of these results in the heart and other benefits in skeletal muscle [97,98,108], a clinical trial was initiated to test the effects of inhibiting phosphodiesterase 5 (PDE5), the enzyme partially responsible for the degradation of cGMP, with sildenafil. Surprisingly, this sildenafil trial was terminated due to several DMD patients receiving sildenafil displaying worsening cardiac function [109]. It is not clear if the sildenafil treatment accelerated the dystrophic disease process or simply failed to show a beneficial effect. Importantly, PDE5 inhibition only prolongs the duration of existing NO signaling, thus it is possible that NO synthesis may be too low or incorrectly localized for sufficient cGMP to be produced in dystrophic cardiomyocytes.

There are several indications that the dystrophic heart has significantly increased levels of direct covalent modification of cysteine residues in the form of S-nitrosylation (SNO). Proteomics-based approaches demonstrate that dystrophic hearts have globally elevated levels of S-nitrosylation, with modifications of the electron transport chain being particularly prominent [110]. Increased S-nitrosylation of the cardiac ryanodine receptor has been shown to increase its Ca^2+^ leak, thus potentially contributing to increases in cytosolic Ca^2+^ and cardiac arrhythmias [58,59]. Furthermore, overexpression of nNOS has been shown to limit fibrosis and improve electrical and contractile properties of the dystrophic heart [111,112,113]. Together, these data describe a very complex role for NO signaling in the dystrophic heart, where it alternates between providing benefit and detriment depending on the particular target or the nature of the study. This complex physiology greatly confounds the use of NO signaling as a therapeutic approach in the dystrophic heart.

### 4.6. Fibrosis

Fibrosis is one of the earliest clinical features of dystrophic cardiac pathology, making its first appearance in DMD patient hearts before 10 years of age [18,114]. Multiple cell types contribute to the development of fibrosis in the heart, including cardiomyocytes, fibroblasts, endothelial cells, and immune cells. All these cells can secrete profibrotic cytokines and chemokines in response to stimuli like inflammation, mechanical signals, and signals from neighboring cells. Fibroblasts and cardiomyocytes are also capable of depositing the extracellular matrix components that comprise fibrotic scars. Fibroblasts are an abundant and reactive cell type in the heart, readily transforming into myofibroblasts and increasing their secretion of ECM and cytokines in response to a large number of endocrine, paracrine, and mechanical signals that include transforming growth factor β (TGFβ), ROS, angiotensin, aldosterone, ECM changes, and many others [115,116]. Cardiomyocytes help drive the fibrotic process by dying and leaving a vacancy that must be filled in with ECM, and also by secreting their own signaling molecules and collagen to contribute to the process [117]. Matrix metalloproteinases (MMPs) and tissue inhibitors of metalloproteinases (TIMPs) modulate the turnover of the deposited ECM through their respective actions of promoting and inhibiting the degradation of matrix components like collagen. Interestingly, there is a trend toward an increase in some MMPs in DMD, with levels of MMP7 in particular significantly correlating with increased cardiac and skeletal muscle pathology in dystrophic patients [118,119]. However, the interpretation of this result is complicated, as the relationship between MMP7 and fibrosis could be secondary to its modulation of inflammation rather than through direct effects on the ECM.

Cellular stress and necrotic death among cardiomyocytes likely kick off fibrosis in the dystrophic heart by polluting the surrounding tissue with cytokines, chemokines, and debris that attract neutrophils and macrophages. These cells are phenotypically diverse, and various contextual factors may contribute to their polarization to promote tissue inflammation or repair through specific paracrine signaling profiles [120]. Furthermore, infiltrating myeloid cells have been shown to harbor aldosterone synthase, thus potentially representing a source of localized aldosterone production and promoting pro-fibrotic gene transcription in mineralocorticoid receptor-expressing fibroblasts and cardiomyocytes [121]. The removal of damaged tissue eventually ends with the deposition of a new fibrotic patch, and these lesions appear on CMR with LGE as areas of bright signal that are often distributed in a sub-epicardial pattern on the left-ventricular free wall [18,20,122]. The high consistency of lesion distribution in a mechanically demanding setting like the heart may indicate that the forces experienced by cardiac cells in this particular region of the heart may be especially injurious, perhaps because of greater cellular dynamics in the epicardium [123].

Aside from the obvious issue of failing to contribute to the pumping action of the heart, accumulation of fibrosis may present additional challenges. First, it likely accelerates further losses of functional myocardium by shifting the workload demand on the neighboring cardiomyocytes, which will now have to carry out excessive contractile work in a stiff, mechanically burdensome environment. Secondly, the scar may interrupt the tracts of electrical conduction through the heart, potentially giving rise to a variety of arrhythmias [7]. Because the fibrotic process starts so early in the disease course and the consequences of unmitigated fibrosis can be so dire, reducing fibrosis has become a major goal of cardiac-directed therapies for DMD. Pharmacological approaches that have shown the capacity to limit the accumulation of fibrosis in dystrophic mouse and patient hearts include ACE inhibitors, ARBs, mineralocorticoid receptor antagonists, and even simvastatin [94,124,125,126].

## 5. Small Molecule Therapies for the Heart

Because of a lack of any potentially curative therapies for dystrophic cardiomyopathy until very recently, these established small molecule-based therapies are aimed at reducing the symptoms and hindering the mechanisms of disease progression in the heart. The four groups of cardiac small molecule approaches listed here are all currently in use in DMD clinical management.

### 5.1. Angiotensin-Inhibiting Therapies

Inhibition of the renin-angiotensin system has become a cornerstone of cardiac-directed therapeutic efforts in DMD patients, with the goal of ameliorating the adverse remodeling that follows cardiomyocyte loss. Angiotensin II (AngII) and the AngII type 1 receptor (AT_1_R) exert many detrimental effects on the heart, including promoting fibrosis, remodeling, ROS production, and cardiomyocyte death [89,92,127,128,129]. Angiotensin receptor blockers (ARBs) and angiotensin converting enzyme inhibitors (ACEIs) are the two main drug classes used to suppress the deleterious effects of angiotensin in heart failure patients in general and in DMD patients specifically. Current guidelines recommend that DMD patients be placed on either one of these two drugs by age 10 or earlier, influenced by the growing body of work reporting the first signs of ongoing cardiac disease processes around 6-10 years of age [18,19,21,22,114]. ACEIs are an older drug class that is often used as the first line of therapy for general heart failure, and was the first to be used in trials to demonstrate improved cardiac function and survival among DMD patients [130,131]. For these reasons, they have often been named first in guidelines and recommendations regarding management of DMD cardiomyopathy and are more frequently prescribed [5,6,132,133]. ARBs are listed as a secondary option or an alternative in cases of poor ACEI tolerance, despite the fact that ARBs are equally effective when compared directly to ACEIs and better tolerated by patients, promoting improved compliance [134,135,136].

Importantly, recent preclinical work has shown that ARBs may have a unique ability to prevent cardiomyocyte damage during acute bouts of increased workload in the heart. Dystrophic mdx mouse hearts displayed 3-fold higher myocardial injury area than wild type hearts after a bolus of the β-adrenergic agonist isoproterenol, but this difference was eliminated by treatment with the ARB losartan [31]. Losartan reduced cardiac damage 2.8-fold in mdx hearts without any significant effect on injury in wild type hearts. Surprisingly, neither acute nor chronic ACEI treatment had any effect on the extent of damage in the mouse heart, suggesting that direct blockade of AT_1_R may be necessary to achieve the full benefit of anti-angiotensin therapy in the dystrophic heart [133]. One potential explanation could be rooted in the fact that ACE is not strictly necessary for the production of AngII and activation of AT_1_R. AngII can be produced through the actions of other peptidases, most notably chymase, thus the inhibition of ACE does not eliminate AngII production [137,138,139]. Furthermore, AT_1_R displays basal activity and can be readily activated further by stretch independently of any ligand, thus potentially giving rise to AngII-independent pathogenic signaling [140,141]. Another compelling explanation for the disparity in benefits of ARBs and ACEIs is the indirect augmentation of cardioprotective AngII type 2 receptor (AT_2_R) signaling as a consequence of AT_1_R blockade [127,142]. AngII is the predominant endogenous ligand for both receptors, and the blockade of AT_1_R feeds back to increase AngII production, resulting in the redirection of a very robust AngII pool for AT_2_R activation [139,142,143].

Further clinical trials with younger patients (<10 years old) will be needed to determine if ARBs show the same unique capacity to preserve cardiomyocytes in the face of ongoing physiological stresses in human dystrophic hearts as they did in the mouse model. Until this work can be carried out, patients with DMD should continue to be prescribed either one of these anti-angiotensin drugs at the earliest age that the families and physicians can agree upon in order to best protect these vulnerable hearts from the permanent loss of myocardial tissue.

### 5.2. Beta-Adrenergic Receptor Blockers

Tachycardia is a common characteristic of DMD, reflecting autonomic dysfunction in the dystrophic heart that predisposes it to arrhythmias. Holter monitoring in DMD patients routinely reveals average heart rates >100 beats per minute, reflecting increased activation of the sympathetic nervous system and increased adrenergic signaling in the heart [6,20,144]. The heart expresses an abundance of β-adrenergic receptors (β-ΑR) in the pacemaker cells, conduction system, and the rest of the myocardium. Activation of cardiac β-AR drives heart rate elevations and increased contractility by augmenting and accelerating calcium transients in the myocyte, which contributes to arrhythmogenesis [145]. Beta-blockers are regularly used for the treatment of acquired heart failure, where they are often combined with ACEIs and diuretics to improve survival and reduce hospitalization rates [146]. Accordingly, beta-blockers have been considered a candidate for cardiac-directed therapy in DMD with the goal of limiting β-AR effects, although it remains unclear whether these drugs deliver a significant benefit that cannot be achieved without them. Investigations using beta-blockers in DMD cardiomyopathy have turned up mixed results, with some studies demonstrating a clear benefit while others show little effect of β-AR blockade on outcomes [144,147,148]. Although some DMD patient studies indicate that beta-blockers preserve cardiac function and survival beyond the effects of ACEIs alone, a recent trial revealed no difference between treatment groups receiving ACEIs alone versus ACEIs with beta-blockers when the ACEI dose was adjusted according to the severity of cardiac dysfunction [144,147,148]. These inconclusive results have contributed to variable and often delayed initiation of beta-blocker use in DMD.

Because beta-blockers are a second-line therapy in DMD, a major obstacle to successfully determining their capacity for benefitting the dystrophic heart may lie in the delayed initiation of therapy and combined use of beta-blockers with first-line drugs. Indeed, it seems most common in clinical practice to start beta-blockers once tachycardia or other arrhythmias become detectable, which may be well after the onset of early cardiac disease processes [6]. Because ACEIs and ARBs continue to be the first line of therapy, it is unlikely that studies investigating the effects of beta-blockers alone on the natural course of the disease will be possible [5]. However, sustained efforts should be made to evaluate outcomes in patients receiving early intervention with beta-blockers compared to those receiving similarly early therapy without beta-blockers in order to identify any significant benefits specific to beta-blocker use.

### 5.3. Mineralocorticoid Receptor Antagonists

Mineralocorticoid receptor (MR) activation by aldosterone can contribute to cardiac pathology by promoting cardiomyocyte death, hypertrophy, and fibrosis in DMD cardiomyopathy and other types of heart failure [149]. For this reason, MR antagonists like eplerenone and spironolactone are a part of the standard approach for managing heart failure with low EF, and are occasionally incorporated into treatment for DMD cardiomyopathy [7]. The potential importance of aldosterone signaling is underscored by reports that myeloid cells infiltrating the site of heart and muscle injury in dystrophic mice express aldosterone synthase, serving as a local source of paracrine aldosterone signaling that may exacerbate the injury and the adverse remodeling that follows it [121]. MR signaling may also play a larger role in DMD due to the ability of corticosteroids like prednisolone, which are used to preserve muscle function in DMD patients, to activate MR in addition to their target receptor [125]. It is worth noting that spironolactone is slightly more potent but has lower selectivity for MR over androgen receptor, resulting in higher potential for side effects associated with androgen imbalance, whereas eplerenone is less likely to interfere with androgen receptor.

Preclinical work using mouse models of DMD cardiomyopathy has shown a protective effect of the combination of spironolactone and ACEI in dystrophic muscles and hearts that was attenuated in the group treated later in life, again reinforcing the importance of proactive initiation of therapy [122,150]. Clinical trials went on to demonstrate that patients with DMD and preserved ejection fraction already receiving treatment with an ACEI or ARB showed modest but significant improvements in myocardial strain, ejection fraction, and chamber dilation with eplerenone treatment, compared to those without eplerenone [149]. The open label extension in patients using eplerenone with ACEI or ARB showed that myocardial circumferential strain was preserved over a period of 36 months [151]. The recent discovery of vamorolone, an MR antagonist that is equally potent to eplerenone and also mirrors the anti-inflammatory benefits of corticosteroids, may represent a critical step toward the inclusion of this MR antagonist as a standard and proactive therapy for DMD cardiomyopathy [125].

### 5.4. Corticosteroids

Currently, a daily regimen with a corticosteroid like prednisone or deflazacort is the earliest and most widely used DMD therapy, based on the ability of steroids to reduce inflammation and prolong strength and ambulation in patients with DMD [7,50,152]. Because steroids are unequivocally beneficial in ameliorating the natural course of DMD in skeletal muscle, therapy is often initiated fairly proactively around 2–5 years of age, making corticosteroids the earliest pharmacological influence on the dystrophic heart [9]. The use of these drugs is not without controversy, with the primary obstacle rooted in challenging systemic side effects, which arise from the classic transcriptional effects of glucocorticoid receptor and include reduced bone density, increased adiposity, and increased muscle catabolism [153]. However, some additional concerns stem from preclinical studies providing evidence that steroids may worsen the progression of DMD in the mouse heart [150,154]. Nevertheless, DMD patient studies are largely in agreement that steroids are likely to be protective in the dystrophic heart, with possible benefits including improved survival, preserved ventricular function, and even reductions in fibrosis [155,156,157,158,159,160]. Perhaps the most telling is the observation that increased survival among the steroid-treated cohort of one study was largely reflective of a striking reduction in heart failure deaths [155].

Vamorolone, the novel drug that recapitulates the anti-inflammatory actions of glucocorticoids and the anti-remodeling effects of MR antagonists, may deliver the same benefits derived from corticosteroid use with minimized side effects [125,161]. It represents a new class of steroids called dissociative steroids, which serve the function of activating glucocorticoid receptor for signaling transduction, but do not result in its binding to glucocorticoid response elements (GRE) and activation of GRE-dependent transcription [153]. It remains to be seen if Vamorolone may replace glucocorticoids and/or MR antagonists in the clinic, but it has already demonstrated efficacy in preclinical studies using dystrophic mice and is currently being tested in DMD patient trials [125,161,162].

## 6. Gene-Targeted Therapies

In recent years, potentially curative approaches for DMD have begun to take shape. Unlike the small molecule treatments, these gene-targeted therapies are designed with the goal to induce expression of a functional gene product that will reestablish normal myocyte physiology. Since these therapies aim to repair the original defect in the cell by restoring functional dystrophin expression, their success is less dependent upon making the correct assumptions regarding the exact mechanisms driving the cellular dysfunction in the absence of dystrophin. However, the high complexity of these gene-targeted systems results in great challenges to achieving efficient and lasting correction. Because of the large variety of mutations that cause DMD, many of these approaches need to be tailored to suit subgroups of patients based on the type and location of their disease-causing mutation (Table 1).

An additional challenge lies in the difficulty of assessing the cardiac effects of these approaches because their efficacy will depend absolutely upon the whole-body efficiency of transduction and dystrophin restoration. Ideally, these corrective therapies should be initiated as early in life as possible, both to achieve maximal preservation of healthy muscle tissue and to facilitate dosing. Since the heart cannot be routinely biopsied to check the efficiency of dystrophin re-expression, clinicians will depend on more sensitive measures like myocardial strain to evaluate patient hearts at early time points in the natural course of the disease. Achieving sufficient therapeutic efficacy in the heart will be of the utmost importance, because correcting skeletal muscle without effectively addressing the cardiomyopathy may result in increased demand on the fragile myocardium, accelerating the progression of heart failure. Even if high efficiency of these therapies is achieved in skeletal muscle, the phenotypic outcome in DMD patients may resemble the disease characteristics of Becker muscular dystrophy or X-linked dilated cardiomyopathy (XLCM) due to the reduced expression and/or truncation of dystrophin. Patients with BMD and XLCM often have sufficient dystrophin expression to display mild skeletal muscle pathology, but BMD has a high risk of heart failure, and XLCM patients succumb to heart failure between 10 and 20 years of age [7,163,164,165,166]. To minimize the risk of this outcome, careful ongoing cardiac monitoring and therapy will be necessary to characterize the cardiac effects of these therapies and successfully manage all aspects of DMD patient health in the context of partial dystrophin replacement. Even DMD patients who undergo treatments with good efficacy in the heart will likely continue to require careful cardiac monitoring and be good candidates for small molecule-based cardiac therapies to limit the effects of improved skeletal muscle function on the dystrophic heart.

### 6.1. Stop Codon Readthrough

Roughly 10% of patients with DMD have a nonsense mutation that replaces a regular amino acid triplet with a premature stop codon and causes the ribosome to terminate translation, failing to synthesize the remainder of the protein [8]. The principle of stop codon read-through, also referred to as nonsense suppression, is simply to induce the ribosome to continue translating the mRNA through the premature stop codon and the rest of the transcript. This concept was initially demonstrated using the aminoglycoside antibiotic gentamicin, but due to the toxicity of gentamicin, ataluren (Translarna) was developed as a safer alternative serving the same purpose. The drug works by binding to the ribosome itself and mildly disrupting its ability to recognize the premature stop codon, enabling the occasional addition of an amino acid in its place, followed by the synthesis of the remainder of the gene [167]. This mechanism appears to be fairly specific to premature stop codons, without allowing readthrough of regular stop codons, and effectively enables partial dystrophin expression in skeletal muscle [167,168].

Based on this preclinical efficacy, ataluren advanced to clinical trials and was approved for the treatment of DMD in Europe, although it is still under investigation for efficacy in the US. Unfortunately, very little evidence exists to inform the question whether ataluren displays any degree of efficiency or delivers a benefit in the heart. Preclinical work suggests that ataluren can induce a modest increase in diffuse dystrophin expression in the mdx mouse heart [168]. Results in human patients are more ambiguous, showing no measurable effect on cardiac function in a small cohort of non-ambulatory DMD patients, although the cardiac function remained stable during the treatment period of 20–24 months [169]. Additional work will need to be performed to better understand the effects of ataluren in the heart and the clinical relevance of these observations.

### 6.2. Antisense-Mediated Exon Skipping

Deletions are the most common type of DMD mutation, with many of them causing dystrophin loss due to the disruption of the open reading frame. When the open reading frame is shifted by a deletion or insertion, it often results in a downstream premature stop codon that terminates translation, so the premise of exon skipping is to sacrifice one exon to restore the open reading frame of the rest of the gene. To accomplish this, antisense oligonucleotides (AON) are designed to bind to the premature mRNA at the splice acceptor site of the mutated exon to obscure the splice site. This causes the spliceosome to move on to the next available splice acceptor site, resulting in the omission of the mutated exon from the final mRNA transcript. Multiple AONs can be used to skip adjacent exons in cases where the removal of multiple exons is required to restore the reading frame. The ultimate goal of this approach in patient with frameshift deletions is the synthesis of a truncated dystrophin protein that still harbors a deletion but retains most of its functionality. Over half of all DMD mutations and up to 80% of deletions are amenable to exon skipping therapy, but since each therapy must be tailored to the mutation region, each successfully developed AON will only rescue a fraction of those mutations [8].

Preclinical studies demonstrated the strong potential of AON to reestablish dystrophin expression in skeletal muscles, but raised concerns about efficacy in the heart based on work showing negligible effects in cardiac muscle [170,171]. Upon probing the underlying cause of this discrepancy, it was found that these AON therapies, often delivered in the form of a phosphorodiamidate morpholino oligomer (PMO), were more likely to be trapped in endosomes in cardiomyocytes than in skeletal muscle [172]. Subsequent preclinical work demonstrated that optimizing the dose and modifying the delivery system could solve this problem in the heart, showing improved cardiac muscle dystrophin restoration with these approaches in dystrophic mice and dogs [173,174]. Currently, conjugation of PMOs to peptides that enhances cell permeability is an active area of development, with preclinical studies demonstrating considerable expression of dystrophin in the hearts of mdx mice and dystrophic dogs treated with high doses of these peptide-conjugated PMO (PPMO) therapies [174,175,176].

At this time, one exon-skipping therapeutic is FDA approved, and several more are in development to target the exons that represent the highest proportions of deletions amenable to exon-skipping. Eteplirsen is a PMO from Sarepta Therapeutics that induces the skipping of DMD exon 51, which may target up to 14% of all mutations. It received FDA approval in 2016 amid controversy, with modest but positive results based on clinical trials and patient experiences despite surprisingly modest effects on dystrophin expression in patient biopsies [177,178]. However, tissue penetration and longevity of PMOs has remained an ongoing concern, and no evidence exists of significant eteplirsen uptake or benefits in the heart [50,179]. To address these shortcomings, other delivery systems for AON are currently being investigated to identify the modifications that can optimize cellular penetration and systemic safety, with a big focus on PPMOs [172]. Additionally, multiple other exon-skipping AONs are being actively developed, with PMOs targeting exons 45 and 53 already in clinical trials. Because these agents only work at the level of premature RNA and have limited longevity in the cell, it will be important to set appropriate guidelines for dosing frequency to reflect sufficient efficacy in the heart in order to minimize the risk of XLCM-like heart failure.

### 6.3. Micro-dystrophin Viral Gene Therapy

Viral gene therapy has long been considered the potential key to curing genetic diseases, based on its theoretical capability to completely replace the missing gene product. For this reason, viral gene replacement therapy is the only approach currently in development for clinical use that offers hope for all DMD patients regardless of the underlying dystrophin mutation and is the only available therapy for patients with mutations that span critically important regions of dystrophin. There is a long history of evidence from animal and human studies highlighting both the great promise and the significant challenges of viral gene therapy, but the field has made major strides toward clinical applications in recent years. The broad lessons learned from this vast body of work are 1) viral gene therapy is feasible and can even be efficiently systemically delivered, 2) only adeno-associated virus (AAV) is currently safe enough to use as a gene therapy vehicle in human patients, and 3) the safety and efficiency of viral gene therapy depend upon many factors that are unique to particular species and also the individual biology of the patient.

In addition to the general limitations of viral gene delivery, the pursuit of a DMD gene therapy faces the additional obstacles of gene size, as the full coding *DMD* gene sequence exceeds the packaging capacity of AAV by over 2-fold. Because the 11 kb cDNA cannot be delivered by an AAV with a 4–5 kb capacity, great effort has been directed at optimally miniaturizing the *DMD* gene by reducing it to its most vital components, producing the candidate micro-dystrophin constructs currently under investigation [180]. Preclinical pursuit of over 30 of these micro-genes largely followed the lessons learned from mildly affected BMD patients with extensive *DMD* sequence deletions, preserving the actin-binding N-terminus and the DGC-binding cysteine-rich domain, but sacrificing large sections of the central rod domain that spans between them [181]. While most of the preclinical work with AAV-delivered micro-dystrophins has focused on skeletal muscle, strong evidence of the cardiac benefits of systemic treatment includes marked reductions in histopathology and improvements in function, supporting their advancement into clinical trials [29,182,183].

Three different micro-dystrophins are currently under investigation in human DMD patient trials of AAV-mediated micro-dystrophin therapy. The composition of the micro-dystrophin constructs in these trials is relatively similar, however the micro-dystrophin vector from Pfizer uses a skeletal muscle promoter that does not drive any expression in the heart [184]. The other two micro-dystrophin AAV trials, spearheaded by Sarepta Therapeutics and Solid Biosciences, are using vectors driven by different promoters that induce expression in both skeletal and cardiac muscle, delivered by a different AAV serotype, and injected at different doses, significantly complicating any comparison between these two trials [181]. Nonetheless, both the MHCK7 promoter in Sarepta’s AAV construct and the CK8 promoter in Solid’s AAV construct have been shown to drive robust cardiac expression of micro-dystrophins used in preclinical studies [185,186].

It is still too soon to know if human cardiac function will be preserved by these micro-dystrophin treatments, but it is possible that these initial trials may reveal the need for higher doses to effectively transduce the majority of the post-mitotic myocardium, and these higher doses may be accompanied by significant side-effects [181]. While some preclinical literature supports the notion that transduction of half of the heart may be sufficient to reap the full benefits, it is difficult to extrapolate confidently from mice to human patients in light of the vastly different body sizes, lifespans, and phenotype severity [32]. Furthermore, AAV-delivered plasmids are not expected to last forever, so repeated dosing (along with immunosuppressive measures like plasmapheresis) may be needed if reductions in the therapeutic effect are detected [187,188]. Finally, even if it becomes possible to achieve complete cardiac transduction and lasting expression of these DMD micro-genes, the treated patient population will still be at a similar risk for developing cardiomyopathy as BMD and XLCM patients due to the extensive truncation of the gene. These limitations on the maximal theoretical efficacy of AAV gene therapy for DMD may result in the continued need for additional small molecule-based management of cardiomyopathy in the setting of an otherwise mild BMD-like phenotype.

### 6.4. CRISPR-Cas9 Gene Editing

CRISPR-Cas9 is a highly versatile genome-editing approach that was found at the root of a bacterial self-defense system against viral infection and gave rise to a promising new strategy for correcting genetic diseases. This system is based simply upon a single guide RNA (sgRNA) molecule that recognizes a genomic sequence and flags down an endonuclease to create a double-stranded break in the DNA at specific recognition sites. In bacteria, both the Cas9 endonuclease and the guide RNAs are encoded by genomic sequences, the latter of which is made up of clustered regularly interspaced short palindromic repeats (CRISPR), but in eukaryotic applications, the sgRNA and Cas9 gene must be delivered exogenously. In non-proliferating cells like skeletal muscle fibers and cardiomyocytes, CRISPR-Cas9 editing can be used to excise a duplication or out-of-frame exon, disrupt a splice site to induce exon skipping, edit a nucleotide in a nonsense mutation, or reframe an exon with a base insertion or deletion that accompanies non-homologous end-joining repair [189,190]. Because the editing occurs at the genomic level and myocyte nuclei will not divide further, this approach represents the potential to treat DMD by reinstating dystrophin expression through a single administration of the therapeutic agent.

Since the CRISPR-Cas9 treatment strategy depends upon the systemic delivery and expression of foreign genes, this therapeutic approach has often made use of AAV-mediated gene delivery in preclinical work to achieve sufficient transduction in vivo, with promising outcomes from a large number of separate studies [190]. Using either a single vector or two separate vectors to encode the sgRNA and the Cas9 sequences with a cardiac-expressing promoter, several studies have demonstrated significant restoration of cardiac dystrophin expression and improvement in cardiac pathology in different mouse and dog models of DMD [191,192,193,194]. Importantly, the correction was shown to persist long-term in mice, supporting the notion of permanent gene repair with this genomic editing strategy [195,196,197].

Despite significant promise, this approach is not without its own setbacks. AAV-mediated gene delivery faces the challenge of possible immunogenicity, and the delivery of a foreign gene exacerbates the issue with the potential of eliciting an immune response to the bacterial protein. Careful screening and immunosuppressive measures will be required to ensure patient safety and effective transduction, although long-term studies in preclinical models suggest immune response against Cas9 does not limit the therapeutic efficacy [191,198]. Furthermore, the possibility of off-target mutagenesis with CRISPR-Cas9 is a cause for concern that has previously been substantiated in human cells, and recent studies have identified dozens of off-target mutations throughout the mouse genome following eight weeks of CRISPR-Cas9 expression [197,199]. While most of these off-target mutations are found in introns, many are in exons, indicating a need for additional preclinical work to investigate the rate of accumulation and the physiological significance of these mutations. The risk of off-target editing can be managed by optimizing both the guide RNAs and the Cas9 endonucleases to minimize non-specific activity and improve fidelity [190,200]. Finally, as with AON-mediated exon skipping, some of the patients undergoing CRISPR-Cas9-mediated exon skipping therapy may still find themselves with a BMD-like risk of cardiomyopathy. Additional preclinical studies with longer post-treatment monitoring in dog and/or non-human primate DMD models will be required to confirm the safety and efficacy of this genome-editing therapy, but current results inspire optimism about the possibility of CRISPR-Cas9 treatment reaching clinical trials.

## 7. Conclusions

Just over 30 years since the discovery of the protein dystrophin, and nearly two centuries after the first descriptions of the devastating disease precipitated by its loss, efforts to understand and correct the underlying pathology of DMD are advancing by leaps and bounds. The preceding decades have been marked by careful mechanistic studies aiming to tease apart the molecular processes derailed by dystrophin loss, providing extensive insights into the cellular dysfunction driving the progressive deterioration and premature death seen in patients. These insights helped to form the foundation for devising symptomatic interventions and small molecule treatment strategies to prolong and improve the lives of patients with DMD. Now, the tireless and innovative efforts of the neuromuscular disease research community have started to open the door for potentially curative approaches that may allow individuals with DMD-causing mutations to live out their adult lives with manageable health.

This turning point in the development of DMD therapies marks the beginning of a new mission to map the shifting landscape of cardiac health and disease in patients that undergo these cutting-edge treatments. This will require continued improvements in cardiac diagnostic and treatment strategies used in the clinics and in preclinical studies that will build upon recent breakthroughs. Deletions are the most common mutation type underlying DMD, but even at their highest theoretical efficiency, these corrective approaches cannot restore missing portions of the DMD gene sequence. For this reason, even patients that are successfully treated with the next generation of gene-targeted therapies will likely continue to benefit from regular monitoring or treatment by a cardiologist. Ensuring that the vulnerable hearts of DMD patients receive careful consideration should remain a major priority in the management of this deadly disease as new questions and possibilities continue to arise from current and future discoveries.

## Figures and Tables

**Table 1:** Summary of currently evolving gene-targeted therapies for inducing dystrophin expression.

Approach	Target Mutation Type	Dystrophin Product	Strengths	Challenges
Stop codon readthrough	Nonsense point mutations	Complete	• Well-tolerated (ataluren)	• *Low efficiency in the heart* • Low prevalence of amenable mutations • Frequent re-dosing
AON-mediated exon skipping ^1^	Frameshift mutations (each AON drug targets one exon)	Lacking existing deletion and additional exon(s)	• Well-tolerated • *Effective at cell level*	• Poor cardiac uptake of PMO^3^ • Frequent re-dosing • Low number of amenable mutations for each AON drug
AAV micro-dystrophin ^2^	Any (does not interact with endogenous gene)	Extensively truncated but functional	• *High efficacy in heart* • High efficacy in skeletal muscle • Lasting (multiple years)	• Potentially immunogenic • Potential for null effect with pre-existing immunity
CRISPR-Cas9	Frameshift, insertion, and nonsense mutations (each sgRNA targets one exon)	Depends on editing strategy (ranging from complete to lacking deletion and additional exon(s))	• *High efficacy in heart and skeletal muscle* • Versatile • Genomic correction is life-long (theoretically)	• Potentially immunogenic • *Risk of off-target editing* • Low number of amenable mutations for each CRISPR drug

^1^ AON—antisense oligonucleotide; ^2^ AAV—adeno-associated virus; ^3^ PMO—phosphorodiamidate morpholino oligomer. Italicized points are based on preclinical studies due to lack of pertinent data in patients.

## Abbreviations

**DMD**	Duchenne muscular dystrophy
**BMD**	Becker muscular dystrophy
**DGC**	Dystrophin glycoprotein complex
**ACEI**	Angiotensin converting enzyme inhibitor
**ARB**	Angiotensin receptor blocker
**RNA**	Ribonucleic acid
**CK**	Creatine kinase
**CMR**	Cardiac magnetic resonance
**LGE**	Late gadolinium enhancement
**MRI**	Magnetic resonance imaging
**cTn**	Cardiac troponin
**ECM**	Extracellular matrix
**IgG**	Immunoglobulin
**LGMD2B**	Limb girdle muscular dystrophy 2B
**LTCC**	L-type calcium channel
**TRP**	Transient receptor potential
**RyR**	Ryanodine receptor
**SR**	Sarcoplasmic reticulum
**SERCA**	Sarcoplasmic/endoplasmic reticulum calcium ATP-ase
**mPTP**	Mitochondrial permeability transition pore
**PCr**	Phosphocreatine
**ATP**	Adenosine triphosphate
**ADP**	Adenosine diphosphate
**ROS**	Reactive oxygen species
**NOX**	NADPH oxidase
**AT_1_R**	Angiotensin II type 1 receptor
**NOS**	Nitric oxide synthase
**AngII**	Angiotensin II
**AT_2_R**	Angiotensin II type 2 receptor
**MR**	Mineralocorticoid receptor
**EF**	Ejection fraction
**XLCM**	X-linked dilated cardiomyopathy
**AON**	Antisense oligonucleotide
**PMO**	Phosphorodiamidate morpholino oligomer
**PPMO**	Peptide-conjugated PMO
**AAV**	Adeno-associated virus
**CRISPR**	Clustered regularly interspaced short palindromic repeats
